# The evolution of labile traits in sex‐ and age‐structured populations

**DOI:** 10.1111/1365-2656.12483

**Published:** 2016-02-22

**Authors:** Dylan Z. Childs, Ben C. Sheldon, Mark Rees

**Affiliations:** ^1^ Department of Animal and Plant SciencesUniversity of SheffieldWestern BankSheffieldS10 2TNUK; ^2^ Department of ZoologyThe Edward Grey InstituteTinbergen BuildingSouth Parks RoadOxfordOX1 3PSUK

**Keywords:** integral projection model, labile trait, ontogeny, *Parus major*, plasticity, Price equation, quantitative trait, quantitative genetics, selection analysis

## Abstract

1. Many quantitative traits are labile (e.g. somatic growth rate, reproductive timing and investment), varying over the life cycle as a result of behavioural adaptation, developmental processes and plastic responses to the environment. At the population level, selection can alter the distribution of such traits across age classes and among generations. Despite a growing body of theoretical research exploring the evolutionary dynamics of labile traits, a data‐driven framework for incorporating such traits into demographic models has not yet been developed.
2. Integral projection models (IPMs) are increasingly being used to understand the interplay between changes in labile characters, life histories and population dynamics. One limitation of the IPM approach is that it relies on phenotypic associations between parents and offspring traits to capture inheritance. However, it is well‐established that many different processes may drive these associations, and currently, no clear consensus has emerged on how to model micro‐evolutionary dynamics in an IPM framework.
3. We show how to embed quantitative genetic models of inheritance of labile traits into age‐structured, two‐sex models that resemble standard IPMs. Commonly used statistical tools such as GLMs and their mixed model counterparts can then be used for model parameterization. We illustrate the methodology through development of a simple model of egg‐laying date evolution, parameterized using data from a population of Great tits (*Parus major*).
4. We demonstrate how our framework can be used to project the joint dynamics of species' traits and population density. We then develop a simple extension of the age‐structured Price equation (ASPE) for two‐sex populations, and apply this to examine the age‐specific contributions of different processes to change in the mean phenotype and breeding value.
5. The data‐driven framework we outline here has the potential to facilitate greater insight into the nature of selection and its consequences in settings where focal traits vary over the lifetime through ontogeny, behavioural adaptation and phenotypic plasticity, as well as providing a potential bridge between theoretical and empirical studies of labile trait variation.

## Introduction

Labile traits are common in animal populations. In contrast to non‐labile traits, which remain constant once they have been expressed, a labile trait is one that is adjusted continuously over the course of an individual's lifetime (Scheiner 1993). Many physiological and behavioural characteristics – such as somatic growth rate and the seasonal timing of reproduction – exhibit reversible development, resulting in a labile phenotype. These kinds of developmental and behavioural changes may represent a form of adaptive plasticity, which has evolved in response to anticipated, short‐term environmental fluctuations (Lande 2014, 2015). Alternatively, context‐dependent constraints on physiology or behaviour may filter variable environmental conditions to generate labile trait variation. Evaluating the adaptive significance of this variation in natural populations is challenging because: (i) observed trait variation may reflect both adaptation and constraint, (ii) individual performance may vary as a consequence of many factors (e.g. sex and age), and (iii) labile trait variation impacts vital rates directly and through its effect on key life‐history events such as maturation.

Integral projections models (IPMs) have been widely adopted in population and evolutionary ecology to derive population‐scale processes from knowledge of continuous, individual‐level state variables (Easterling, Ellner & Dixon 2000; Rees, Childs & Ellner 2014; Griffith *et al*. 2016). These variables are typically labile, in the sense that they vary over the course of an individual's lifetime, driving patterns of variation in vital rates and life histories (Plard *et al*. 2016). Although the majority of published IPMs have considered body size, in principle the focal state variables can be any demographically important continuous attribute such as breeding date or territory size. The basic IPM has been extended to incorporate multidimensional states that may include both categorical (e.g. developmental stage, breeding status) and continuous variables (Ellner & Rees 2006). Two important special cases of such ‘complex’ IPMs for many animal populations are sex‐ and age‐structured models (Childs *et al*. 2003; Schindler *et al*. 2013, 2015). Age is a reliable predictor of demographic performance in many populations, although the functional dependence of mortality and reproduction on age is complicated by trait‐mediated effects (Brooks *et al*. 2016) and many other processes (e.g. behavioural adaptation and senescence) that interact to shape these relationships. Age‐structured IPMs provide a powerful framework for understanding how these effects play out over the life cycle and at the population level.

Integral projection models have been applied to address two broad categories of questions in evolutionary demography. The first deals with evolutionary statics; that is, it characterizes evolutionary endpoints. Starting with assumptions about trait‐dependent demography, trade‐offs between vital rates and the action of density dependence, the goal is to predict parameter values that are an evolutionary stable strategy (Dercole & Rinaldi 2008). Several early applications of IPMs used this framework to characterize optimal life‐history traits, including flowering size in plants (Childs *et al*. 2004; Rees *et al*. 2004, 2006; Hesse, Rees & Múeller‐Scháerer 2008), seed germination rates (Rees *et al*. 2006), and twinning frequency in a mammal (Childs *et al*. 2011). The second class of question deals with evolutionary dynamics. Two approaches to this type of question have been adopted. The first forgoes an explicit genetic model, and instead uses phenotypic associations between parents and offspring to subsume inheritance and parental effects into a single ‘inheritance function’. This framework has been promoted for studying the joint dynamics of ecological and evolutionary change [‘eco‐evolutionary’ processes, reviewed in Smallegange & Coulson (2013)]. The second approach embeds explicit assumptions about the genetic basis of a focal trait into an IPM, and then uses the resulting model to simulate short‐term changes in the mean genotype of competing clones [see Rees & Ellner (2016)], and allele frequencies in diploid populations (Coulson *et al*. 2011). However, a framework for accommodating quantitative trait variation has not yet been formalized.

Integral projection models are frequently constructed so that analytical tools from evolutionary demography can be applied to investigate the mechanisms of change predicted by the model (Coulson 2012; Smallegange & Coulson 2013). Coulson & Tuljapurkar (2008) introduced one such tool, the ‘age‐structured Price equation’ (ASPE), to decompose changes in the population‐level mean phenotype of age‐structured populations. This extension of the Price equation (Price 1970) divides fitness into its age‐specific survival and recruitment components, which along with their demographic weights (reflecting the population age structure) are used to partition change in the mean phenotype into contributions resulting from variation in demographic structure, age‐specific selection via differences in survival and recruitment, phenotypic plasticity and growth, and differences between offspring and parental trait values. The ASPE was initially applied directly to observational data. Equivalent model‐based calculations were later derived by Coulson, Tuljapurkar & Childs (2010). The ASPE has been used to examine apparent stasis in birthweight in red deer (Coulson & Tuljapurkar 2008), and body mass dynamics of Soay sheep (Ozgul *et al*. 2009) and yellow‐bellied marmots (Ozgul *et al*. 2010). However, the Price equation and the derived ASPE represent very general decompositions of change that may be applied to any quantity that changes among time intervals, including allele frequencies or breeding values (Frank 1997). Despite its generality, the ASPE has not been used to investigate the dynamics of genetic change in a demographic model.

Here, we describe a mathematical framework to incorporate labile traits with a quantitative genetic underpinning into models of age‐structured, two‐sex populations. The resulting data‐driven modelling framework shares many of the advantages of IPMs. It allows models to be constructed from knowledge of individual‐level processes and permits quantitative genetic parameters – commonly estimated in wild populations using the animal model (Kruuk 2004) – to be incorporated so that microevolutionary dynamics can be predicted under realistic assumptions about age structure, life histories and individual trait–fate relationships. We illustrate the methodology by constructing a simple model of laying date ‘synchrony’, parameterized with data from a population of Great tits (*Parus major*) and then use the model to project change in the synchrony and density of breeding pairs. To understand the behaviour of the model, we develop a simple extension of the ASPE that accommodates two sexes (under certain assumptions), and then apply this decomposition to our model predictions to partition sources of change in the mean laying date phenotype and the breeding values. r scripts for the implementation of this approach are made available on Figshare (Childs 2015).

## Modelling framework

In this section, we outline a general family of discrete time models that project the joint dynamics of a (multivariate) phenotype–genotype distribution and population density. The approach parallels that developed by Barfield, Holt & Gomulkiewicz (2011) to analyse quantitative trait dynamics in stage‐structured populations, but here we consider continuous, labile traits in a sex‐ and age‐structured population subject to a time‐varying environment. We do not prescribe the nature of temporal variation, but the model can accommodate features such as density dependence or a secular trend in vital rates. We assume the infinitesimal model of inheritance (Fisher 1918; Falconer & Mackay 1996) in the following derivations, although alternative genetic models are possible.

### Definitions and notation

We let $x\;=\;(x_{1},x_{2},\ldots ,x_{u})$ denote the continuous, multivariate ‘*l*‐state’ of an individual. The *l*‐state includes any component of the phenotype that varies over the life cycle as a result of ontogeny or phenotypic plasticity; that is, *l*‐states are labile at the individual level. We assume that each component of the *l*‐state influences one or more vital rates, either directly or indirectly via its impact on another component. Examples of possible *l*‐states include body mass or size, morphometric character states, physiological markers of stress, or a measure of reproductive timing such as first egg‐laying date. Individuals are further characterized by a constant ‘*q*‐state’, denoted $z\;=\;(z_{1},z_{2},\ldots ,z_{v})$. The *q*‐state is a quantitative trait assigned at birth, which remains constant over life and influences one or more vital rates, either directly or indirectly via its impact on the dynamics of **x**. Following the usual conventions of quantitative genetics, we let **z** = **g** + **e**, where **g** is the additive genetic value (i.e. the breeding value) and **e** is the permanent environmental deviation. Thus, an individual's current state is uniquely defined by three vectors **x**,** z** and **g**. Their state one time step later is denoted $x^{\prime }$, $z^{\prime }$ and $g^{\prime }$.

In the definitions that follow, we will only use the term *probability density function* (pdf) to refer to functions that ‘sum to on’. A function that describes some general aspect of continuous population structure – but does not possess this property – is called a *density function*. Our model projects (at time *t*) the joint density function of *l*‐ and *q*‐states of female and male of age *a*, denoted $n_{f}^{\left(t\right)}\left(x,z,g,a\right)$ and $n_{m}^{\left(t\right)}\left(x,z,g,a\right)$, respectively. These are defined such that

(eqn 1) $$\begin{array}{cc} N_{f}^{\left(t\right)}\left(a\right) & =\int \int \int n_{f}^{\left(t\right)}\left(x,z,g,a\right)dx\;dz\;dg\\ N_{m}^{\left(t\right)}\left(a\right) & =\int \int \int n_{m}^{\left(t\right)}\left(x,z,g,a\right)dx\;dz\;dg, \end{array}$$

where $N_{f}^{\left(t\right)}\left(a\right)$ and $N_{m}^{\left(t\right)}\left(a\right)$ are the abundance of age *a* females and males at time *t*, respectively (we exclude the integration domain to keep our notation compact; all integrals are over the entire range of all variables). The total population abundance is then $N^{\left(t\right)}\;=\;\sum_{a}\left(N_{f}^{\left(t\right)}\left(a\right)+N_{m}^{\left(t\right)}\left(a\right)\right)$. The demography and trait dynamics are governed by a set of functions (defined below), any of which may vary with time as a result of variation in the external environment or population density. This time dependence and/or density dependence is denoted by the superscript (*t*). Finally, we use the notation ${\left\langle x,z,g,a\right\rangle }_{f}$ and ${\left\langle x,z,g,a\right\rangle }_{m}$ to denote a unique combination of female and male states, and use $h({\left\langle x,z,g,a\right\rangle }_{f})$ as a shorthand for $h(x_{f},z_{f},g_{f},a_{f})$, where *h* is some general function.

### Survival and growth

The dynamics of a cohort are determined by their state‐dependent survival and ‘growth’. We use the term growth generically to refer to any change in the *l*‐state distribution over the life cycle, although the focal trait(s) need not be related to body size or morphology. Our model assumes that only **x**,**z** and *a* determine survival rates and growth dynamics (note that, because **g** is a component of **z**, this means an individual's breeding value can affect vital rates – we discuss this reasoning further in the Parameterization section later). The survival probability of age *a* females and males are described by the survival functions, $s_{f}^{\left(t\right)}\left(x,z,a\right)$ and $s_{m}^{\left(t\right)}\left(x,z,a\right)$, respectively. We write each of these as a single function, although they may subsume more than one process. For example, if reproduction is fatal – such as in certain salmonids – then $s_{f}^{\left(t\right)}\left(\ldots \right)$ will be defined in terms of two functions describing reproduction and mortality due to other processes (Childs *et al*. 2003, 2004). The conditional distributions of female and male *l*‐states next year, $x^{\prime }$, given their current state, are governed by the growth kernels, $G_{f}^{\left(t\right)}\left(x^{\prime }|x,z,a\right)$ and $G_{m}^{\left(t\right)}\left(x^{\prime }|x,z,a\right)$, respectively. As an individual's breeding value does not change as it ages, the density functions of female and male cohorts after one time step are then

(eqn 2) $$\begin{array}{cc} n_{f}^{\left(t+1\right)}\left(x^{\prime },z,g,a+1\right) & =\int s_{f}^{\left(t\right)}\left(x,z,a\right)\\ & G_{f}^{\left(t\right)}\left(x^{\prime }|x,z,a\right)n_{f}^{\left(t\right)}\left(x,z,g,a\right)\;dx\\ \text{and}\;\;n_{m}^{\left(t+1\right)}\left(x^{\prime },z,g,a+1\right) & =\int s_{m}^{\left(t\right)}\left(x,z,a\right)\\ & \;G_{m}^{\left(t\right)}\left(x^{\prime }|x,z,a\right)n_{m}^{\left(t\right)}\left(x,z,g,a\right)\;dx. \end{array}$$

These expressions are equivalent to the survival–growth component of an IPM; an individual must survive to remain in their cohort, and then, their contribution to the labile component of the state distribution is projected from their current state.

### Reproduction

The derivation of the density function of new recruits is less straightforward; the existence of two sexes introduces considerable complexity into population models, and the calculations cannot be written in a completely general form because the details will depend on the biology of the system. We give one example here that applies to an annually breeding species exhibiting biparental care, where stable pairs form once each year. The number of recruits each pair produces and the *l*‐state of these recruits are jointly determined by some combination of the *l*‐ and *q*‐state of their parents. The case study we examine later represents a much simpler, special case of this model. Alternative models that may apply to different settings are discussed in the next section.

Three processes need to be considered to project reproduction. We first need to form the joint density function of breeding pairs, $p^{\left(t\right)}({\left\langle x,z,g,a\right\rangle }_{f},{\left\langle x,z,g,a\right\rangle }_{m})$. This will generally be the most challenging component of a model to develop, as it results from frequency‐ and density‐dependent processes that govern formation of breeding pairs, and may reflect other demographic processes operating on parents. For example, if we aim to project the dynamics of a population from a point in time shortly after reproduction occurs, $n_{f}^{\left(t\right)}\left(\ldots \right)$ and $n_{m}^{\left(t\right)}\left(\ldots \right)$ will be scaled by survival terms; individuals that do not survive until the next breeding attempt cannot contribute recruits. However, it will sometimes be possible to adopt a fairly simple form for $p^{\left(t\right)}\left(\ldots \right)$. For example, if we assume that the number of breeding pairs is strictly limited by the less abundant sex, but that every individual has an equal probability of successfully forming a pair, then

(eqn 3) $$\begin{array}{cc} p^{\left(t\right)}({\left\langle x,z,g,a\right\rangle }_{f},{\left\langle x,z,g,a\right\rangle }_{m}) & =N_{b}^{\left(t\right)}n_{f}^{\left(t\right)}\left(x,z,g,a\right)\\ & \;n_{m}^{\left(t\right)}\left(x,z,g,a\right)/\zeta^{\left(t\right)} \end{array}$$

where $N_{b}^{\left(t\right)}\;=\;\text{min}\left(N_{f}^{\left(t\right)},N_{m}^{\left(t\right)}\right)$, and $\zeta^{\left(t\right)}$ is the normalization constant that converts the product of $n_{f}^{\left(t\right)}\left(\ldots \right)$ and $n_{m}^{\left(t\right)}\left(\ldots \right)$ in this expression into a probability density function (note that $n_{f}^{\left(t\right)}\left(\ldots \right)$ and $n_{m}^{\left(t\right)}\left(\ldots \right)$ are not evaluated at the same values of the **x**,** z**,** g** and *a* arguments in this expression). Where such simplifying assumptions cannot be justified, it will be necessary to construct a ‘marriage function’, that takes arguments $n_{f}^{\left(t\right)}\left(\ldots \right)$ and $n_{m}^{\left(t\right)}\left(\ldots \right)$, and maps these to the density $p^{\left(t\right)}\left(\ldots \right)$ (Schindler *et al*. 2013).

The second two processes describe the production of offspring and their states. Here again, the effect of **g** on offspring number and state (if modelled) plays out indirectly through its contribution to **z**. The number of female and male recruits produced by a pair are described by fertility functions, $b_{\to f}^{\left(t\right)}({\left\langle x,z,a\right\rangle }_{f},{\left\langle x,z,a\right\rangle }_{m})$ and $b_{\to m}^{\left(t\right)}({\left\langle x,z,a\right\rangle }_{f},{\left\langle x,z,a\right\rangle }_{m})$, respectively, which are functions of maternal and paternal states. The distributions of female and male recruit *l*‐states, conditional on the state of their parents, are given by the kernels $C_{\to f}^{\left(t\right)}(x^{\prime }{|\left\langle x,z,a\right\rangle }_{f},{\left\langle x,z,a\right\rangle }_{m})$ and $C_{\to m}^{\left(t\right)}(x^{\prime }{|\left\langle x,z,a\right\rangle }_{f},{\left\langle x,z,a\right\rangle }_{m})$, respectively. In mathematical terms, there is little difference between this kernel and that defined in ‘classic’ IPMs, in the sense that both are just conditional probability density functions of offspring state, where the conditioning is with respect to parental state(s). However, as classic IPMs are only structured by phenotypic traits, and most have only considered a single sex [but see Schindler *et al*. (2013, 2015)], the conditioning has typically been simpler.

The density functions of new female and male recruits, $n_{f}^{\left(t+1\right)}\left(x^{\prime },z^{\prime },g^{\prime },0\right)$ and $n_{m}^{\left(t+1\right)}\left(x^{\prime },z^{\prime },g^{\prime },0\right)$, are calculated in several steps. First, the expected number of new female and male recruits, $N_{f}^{\left(t\;+\;1\right)}\left(0\right)$ and $N_{m}^{\left(t\;+\;1\right)}\left(0\right)$, is calculated as

(eqn 4) $$\begin{array}{cc} N_{f}^{\left(t+1\right)}\left(0\right) & =\sum_{a_{f},a_{m}}\int \int \int \int b_{\to f}^{\left(t\right)}({\left\langle x,z,a\right\rangle }_{f},{\left\langle x,z,a\right\rangle }_{m})\\ & \;p^{\left(t\right)}({\left\langle x,z,a\right\rangle }_{f},{\left\langle x,z,a\right\rangle }_{m})dx_{f}\;dz_{f}\;dx_{m}\;dz_{m}\\ N_{m}^{\left(t+1\right)}\left(0\right) & =\sum_{a_{f},a_{m}}\int \int \int \int b_{\to m}^{\left(t\right)}({\left\langle x,z,a\right\rangle }_{f},{\left\langle x,z,a\right\rangle }_{m})\\ & \;p^{\left(t\right)}({\left\langle x,z,a\right\rangle }_{f},{\left\langle x,z,a\right\rangle }_{m})dx_{f}\;dz_{f}\;dx_{m}\;dz_{m}, \end{array}$$

where $p^{\left(t\right)}({\left\langle x,z,a\right\rangle }_{f},{\left\langle x,z,a\right\rangle }_{m})\;=\;\int \int p^{\left(t\right)}({\left\langle x,z,g,a\right\rangle }_{f},{\left\langle x,z,g,a\right\rangle }_{m})dg_{f}dg_{m}$. We then calculate the joint probability density function of maternal genotype, paternal genotype and recruit *l*‐state among female and male recruits, denoted $\chi_{f}^{\left(t\right)}\left(g_{f},g_{m},x^{\prime }\right)$ and $\chi_{m}^{\left(t\right)}\left(g_{f},g_{m},x^{\prime }\right)$. For females, this is given by

(eqn 5) $$\begin{array}{cc} \chi_{f}^{\left(t\right)}\left(g_{f},g_{m},x^{\prime }\right) & =\frac{1}{N_{f}^{\left(t+1\right)}\left(0\right)}\sum_{a_{f},a_{m}}\int \int \int \int b_{\to f}^{\left(t\right)}({\left\langle x,z,a\right\rangle }_{f},\\ & \;{\left\langle x,z,a\right\rangle }_{m})\times C_{\to f}^{\left(t\right)}(x^{\prime }{|\left\langle x,z,a\right\rangle }_{f},{\left\langle x,z,a\right\rangle }_{m})\\ & \;\times p^{\left(t\right)}({\left\langle x,z,g,a\right\rangle }_{f},{\left\langle x,z,g,a\right\rangle }_{m})\\ & \;dx_{f}\;dz_{f}\;dx_{m}\;dz_{m} \end{array}$$

where $N_{f}^{\left(t+1\right)}\left(0\right)$ normalizes the integrands in equation (eqn 5) to ensure that we are working with probability density functions. For males, the function has the same form. With these pdfs in hand, the next step is to calculate the joint *l*‐ and *q*‐state probability density function of female and male recruits, denoted $\Psi_{f}^{\left(t+1\right)}\left(x^{\prime },z^{\prime },g^{\prime }\right)$ and $\Psi_{m}^{\left(t+1\right)}\left(x^{\prime },z^{\prime },g^{\prime }\right)$, respectively. In brief, because we assume the infinitesimal model of inheritance, we need to compute the mid‐parent distribution of $g_{f}$ and $g_{m}$ in offspring and then add the Gaussian segregation variance to construct these distributions. These calculations are cumbersome, so we relegate them to the (Appendix S1, Supporting information). Finally, to calculate the density function of female and male recruits, $n_{f}^{\left(t+1\right)}\left(x^{\prime },z^{\prime },g^{\prime },0\right)$ and $n_{m}^{\left(t+1\right)}\left(x^{\prime },z^{\prime },g^{\prime },0\right)$, we rescale $\Psi_{f}^{\left(t+1\right)}\left(x^{\prime },z^{\prime },g^{\prime }\right)$ and $\Psi_{m}^{\left(t+1\right)}\left(x^{\prime },z^{\prime },g^{\prime }\right)$ by the number of female and male recruits, respectively, such that $n_{f}^{\left(t\;+\;1\right)}\left(\ldots \right)$ and $n_{m}^{\left(t+1\right)}\left(\ldots \right)$ are

(eqn 6) $$\begin{array}{cc} n_{f}^{\left(t+1\right)}\left(x^{\prime },z^{\prime },g^{\prime },0\right) & =N_{f}^{\left(t+1\right)}\left(0\right)\Psi_{f}^{\left(t+1\right)}\left(x^{\prime },z^{\prime },g^{\prime }\right)\\ \text{and}\;\;n_{m}^{\left(t+1\right)}\left(x^{\prime },z^{\prime },g^{\prime },0\right) & =N_{m}^{\left(t+1\right)}\left(0\right)\Psi_{m}^{\left(t+1\right)}\left(x^{\prime },z^{\prime },g^{\prime }\right). \end{array}$$

### Alternative models

The model implied by equations (eqn 2), (eqn 3), (eqn 4), (eqn 5), (eqn 6) is relatively high dimensional compared to a standard IPM. Even a model with one‐dimensional **x**,** z**, and **g** requires numerical integration to be implemented over four dimensions (equation 5). Standard numerical quadrature routines can be employed to solve a problem of this size, but these will soon become intractable as the dimension of the *l*‐ or *q*‐states is increased. Fortunately, considerable simplification of the model will often be possible. In many populations, it may be reasonable to assume that offspring production and the sex ratio are solely determined by breeding females (‘maternal demographic control’). Under these conditions, parental genotypes do not covary among offspring, which somewhat simplifies the calculations describing genetic transmission across generations. This case is considered in Appendix A.2. Further simplification will be possible if one can also assume that parental effects only operate via females (‘maternal phenotypic control’). Under these conditions, the dimensionality of the reproduction and transmission components of the model is much smaller, which considerably simplifies the model. This case is examined in Appendix A.3. Models that exclude parental effects altogether, such as the case study below, are even simpler to derive and numerically implement.

### Parameterization

Just as with a standard IPM, our model can be parameterized using individually structured, longitudinal data of the kind now routinely collected in many long‐term studies (Clutton‐Brock & Sheldon 2010). A series of regression models may be fitted to such data to describe trait variation over the life cycle, the relationship between parental and offspring trait distributions, and trait–fate relationships, with the additional requirement that sufficient pedigree data must be available to partition genetic variance in a parameter of at least one of these models. It may not always be straightforward to decide *a priori* how to associate a focal trait that has been measured in the laboratory or in the field, with the *l*‐ and *q*‐state components of a model, but an appropriate designation should become apparent once the data have been statistically modelled. In order to understand how to reason about these decisions, we make a distinction between ontogenetic traits that change systematically over the course of development, and plastic traits that vary over the life cycle in response to environmental conditions. We discuss these two possibilities in turn, denoting the focal trait as *y*, to distinguish it from the *l*‐ and *q*‐state variables (*x* and *z*), which are properties of the model rather than the data. We use standard subscript notation to describe statistical models involving these variables – for example $x_{it}$ may denote the size of individual *i* in year *t* – but drop these subscripts when describing the corresponding demographic model components. Finally, for the purpose of simplifying this discussion we only consider univariate focal traits.

When modelling an ontogenetic trait, it will often be natural to define the *l*‐state such that it corresponds directly to the focal trait (i.e., *y* = *x*), and to work with a growth kernel that is similar to those used in a standard IPM. These kernels are usually derived from a non‐stationary, first‐order autoregressive model – that is an antedependence model (Zimmerman & Nunez‐Anton 2009) – that is fitted by regressing successive trait values against one‐another. If we ignore age dependence, the simplest statistical model for the trait of individual *i* is then, $x_{it+1}\;=\;\gamma_{0}\;+\;\gamma_{1}x_{it}+\epsilon_{it}$, where $x_{it}$ is the value in year *t*, $\gamma_{0}$ and $\gamma_{1}$ are regression coefficients, and $\epsilon_{it}$ is a normally distributed *iid* error term. The growth kernel that arises from this model is then, $G^{\left(t\right)}\left(x^{\prime }|\;x\right)\;=\;f_{N}\left(E\left[x^{\prime }|\;x\right],\sigma_{G}^{2}\right)$, where $f_{N}$ is the normal density function, the expected value $E\left[x^{\prime }|\;x\right]\;=\;\gamma_{0}+\gamma_{1}x$, and $\sigma_{G}^{2}$ is the variance in growth. Various extensions to this model have been employed. For example, where there is sufficient temporal replication, the $\gamma_{0}$ and $\gamma_{1}$ coefficients may be allowed to vary by time to capture fluctuating growth conditions. Nonlinear dependence of $x_{it+1}$ on $x_{it}$ might be accommodated by replacing the $\gamma_{1}x_{it}$ term with a flexible smooth function, $g(x_{it})$, fitted using a generalized additive model. Whatever the underlying growth model, the functional dependence of the focal trait on age is not prescribed directly, but is instead a consequence of its distribution at birth, the growth kernel, and – at the population level – the trait‐dependent survival function.

To extend this basic kernel so that it may be used in the modelling framework described here, among‐individual differences can be captured by including one or more normally distributed, individual‐level random effects (Rees *et al*. 2000; Ellner & Rees 2006, 2007; Vindenes & Langangen 2015), and where sufficient pedigree data are available, these variance components may be further partitioned into additive genetic and permanent environment effects. For example, the linear model underlying a varying‐intercept growth model might be $x_{it+1}\;=\;\gamma_{0}\;+\;z_{i}\;+\;\gamma_{1}x_{it}+\epsilon_{it}$, where $z_{i}\;=\;g_{i}\;+\;e_{i}$, such that $g_{i}$ and $e_{i}$ are the additive genetic and permanent environment effects associated with individual *i*. This model may be used as the basis of a growth kernel in our framework, that is $G^{\left(t\right)}\left(x^{\prime }|\;x,z\right)\;=\;f_{N}\left(E\left[x^{\prime }|\;x,z\right],\sigma_{G}^{2}\right)$, where the expected value is now, $E\left[x^{\prime }|\;x,z\right]\;=\;\gamma_{0}+z+\gamma_{1}x$. The *l*‐state (*x*) variable corresponds directly to the trait of interest, while the *q*‐state (*z*) simultaneously captures heritable differences in the *rate of state change*, conditional on current state, and the asymptotic trait an individual may reach; that is, the *q*‐state does not prescribe a trait at any given age, but instead determines the dynamics of the growth increments at each age. Greater flexibility in growth trajectories might be introduced by accommodating among‐individual variation in the trait‐slope, or by allowing the intercept and slope coefficients to vary with time.

When modelling a labile plastic traits that can change repeatedly in life (contrary to plastic traits that take one value during life), it may be reasonable to partition *y* into separate *l*‐ and *q*‐state components. A putative linear model describing its dynamics is $y_{it}\;=\;z_{it}\;+\;x_{it},\;\text{where}x_{it}\;=\;\gamma_{0}\;+\;\gamma_{1}e_{t}\;+\;\epsilon_{it}$. Here, the *l*‐state (*x*) is a function of $\gamma_{0}$ and $\gamma_{1}$ – intercept and slope coefficients that define a reaction norm with respect to an annually varying environmental effect, $e_{t}$ – and the normally distributed *iid* error term, $\epsilon_{it}$. The *q*‐state in this model captures consistent among‐individual differences in the expression of the focal trait, which do not vary over the life cycle. The ‘growth’ kernel corresponding to this model is simply $G^{\left(t\right)}\left(x^{\prime }\right)\;=\;f_{N}\left(E\left[x^{\prime }\right],\sigma_{G}^{2}\right)$, where $E\left[x^{\prime }\right]\;=\;\gamma_{0}+\gamma_{1}e_{t+1}$ and $\sigma_{G}^{2}$ is the residual variance in the trait. Alternative models are possible. In the simple case study below, we consider a plastic trait in which there is no explicit environmental driver of trait variation, but where the *l*‐state component of the focal trait is autocorrelated and the *q*‐state only contains an additive genetic component, *g*.

The distinction between ontogenetic and plastic traits is not concrete. For example, if the intercept and slope coefficients of an antedependence model vary with respect to an explicit environmental covariate, then both ontogenetic and plastic changes in the focal trait can be accommodated by the ‘ontogenetic’ growth kernel described above. The choice of model used to capture variation in the focal trait has implications for how the remaining vital rate functions are parameterized. Functions that describe components of reproduction and survival are typically derived from (G)LMs that include the focal trait as a covariate. For example, if $S_{it}$ is a binary index of annual survival, it may be modelled using a logistic regression as $S_{it}∼Bern\left(E\left[S_{it}\right]\right)$, where $logit\left(E\left[S_{it}\right]\right)\;=\;\gamma_{0}^{\left(t\right)}+\gamma_{1}y_{it}$. If we had directly modelled the dynamics of the focal trait using the ontogenetic model, then *y* = *x*, and the resulting survival function, *s*(*x*), only depends on the *l*‐state. On the other hand, if we had partitioned *y* into *l*‐ and *q*‐state components using the plastic trait model, then *y* = *x* + *z*, and the resulting survival function, *s*(*x*,*z*), depends on both these components of the trait. Note that, in either case, the reproduction and survival functions may depend on additional components of the *q*‐state, which capture permanent among‐individual differences in performance that are not attributed to the focal trait.

## Case study: egg‐laying date synchrony

We now demonstrate an application of our framework to predict the microevolutionary dynamics of breeding phenology, using data from a long‐term study of the Great tit (*Parus major*) population at Wytham Woods, Oxford, UK. This population has exhibited a marked change in breeding date over time (Charmantier *et al*. 2008). The mean egg‐laying date of females and the timing of peak abundance of winter moth (*Operophtera brumata*) larvae – an important food resource for juvenile great tits – have advanced by a similar amount (∼ 2 weeks) since the 1970s. These changes are very well‐matched, such that the average synchronization of laying date with the timing of caterpillar emergence has remained unchanged over the course of the study. This consistent synchronization is thought to be driven by phenotypic plasticity of egg‐laying date in response to temperature increases in the period preceding egg laying, and by the fact that caterpillar timing is also phenotypically plastic and seems to be responding at similar rate, perhaps because driven by similar temperature variation (Charmantier *et al*. 2008).

Nonetheless, although phenotypic plasticity clearly plays a central role in tracking environmental change in the Wytham population, two lines of evidence indicate that the degree of tracking is imperfect: (i) the mean egg‐laying date fluctuates relative to peak winter moth abundance (Van Noordwijk, McCleery & Perrins 1995), resulting in a degree of fluctuating selection, and (ii) phenotypic selection analysis indicates that directional selection for earlier egg‐laying date operates in most years (Charmantier *et al*. 2008). To better understand the ecological and evolutionary consequences of these selective processes, we constructed a model to examine the potential for egg‐laying date *synchrony* to evolve. We define this trait (*S*, ‘laying date synchrony’) to be the difference between egg‐laying date (*L*) and ‘half‐fall date’ (*HFD*), such that *S* = *L*−*HFD*. The half‐fall date (the median date that fifth‐instar winter moth are caught descending from trees to pupate) is a standardized measure of the timing of the peak of larval biomass of winter moth larvae in this population. In the face of reliable, individual adjustment of behaviour in response to the environment, this measure provides a simple phenomenological means of capturing the (mis)match between optimal egg‐laying date and peak food availability. Note that *S* takes negative values; females commence laying approximately 35 days prior to peak winter moth abundance.

### Population model

The population model projects the dynamics of breeding pair density and female laying date synchrony in a temporally stochastic, density‐dependent environment. We model laying date synchrony as a strictly sex‐limited, age‐dependent state variable. For simplicity, we also assume that maternal effects and common environment effects (e.g. nest effects) are absent; that is, a female's laying date synchrony depends on her own genotype. The resulting model is a special case of the model considered earlier. The focal state variable is defined by two univariate, additive components in breeding females: a genetic (and breeding) value, *g*, and an labile component *x*, which corresponds to the individual‐specific deviation from the annual population mean, over and above that due to *g*. We do not include a permanent environment effect. Instead, we assume that successive *x* are autocorrelated across successive ages, within individual females; this autocorrelation term serves as a single proxy for various different environmental sources repeatability in the trait expression (see Supporting information). The realized laying date synchrony is then given by $S\;=\;x\;+\;g\;+\;\gamma_{a}^{\left(t\right)}$, where $\gamma_{a}^{\left(t\right)}$ is the annual, age‐specific deviation from the mean that captures annual fluctuations in the degree of synchrony. The joint density function of age *a* female and male states projected by the model are denoted $n_{f}^{\left(t\right)}\left(x,g,a\right)$ and $n_{m}^{\left(t\right)}\left(g,a\right)$, respectively. Notice that, because we assume the absence of a permanent environment effect, *z* = *g*, we can denote the arguments of functions in the model as (*x*,*g*,*a*), rather than (*x*,*z*,*a*).

The survival probability of age *a* females is denoted $s_{f}^{\left(t\right)}\left(x,g,a\right)$, and the conditional distribution of the females’ labile component of laying date, given their current state, is governed by the age‐dependent kernel $G_{f}^{\left(t\right)}\left(x^{\prime }|\;x,a\right)$. The density function of a female cohort after one time step is then

(eqn 7) $$n_{f}^{\left(t+1\right)}\left(x^{\prime },g,a+1\right)=\int s_{f}^{\left(t\right)}\left(x,g,a\right)G_{f}^{\left(t\right)}\left(x^{\prime }|\;x,a\right)n_{f}^{\left(t\right)}\left(x,g,a\right)\;dx.$$

The survival function, $s_{f}^{\left(t\right)}\left(x,g,a\right)$, is derived from a logistic regression. The model includes second‐degree polynomial terms for laying date synchrony and age, and linear breeding pair density and secular trend terms. The resulting $s_{f}^{\left(t\right)}\left(x,g,a\right)$ is density‐dependent, but we fix the secular trend effect to the value acting in the middle of study period. The coefficients associated with the laying date terms in the survival model are allowed to vary among years to accommodate fluctuations in mean survival and selection. The ’growth’ kernel for the labile component of laying date synchrony, $G_{f}^{\left(t\right)}\left(x^{\prime }|\;x,a\right)$, is modelled as a normal density function with conditional mean given by $\mu_{G}\;=\;\rho x+\gamma_{2}^{\left(t+1\right)}$, where *ρ* captures the autocorrelation and $\gamma_{2}^{\left(t+1\right)}$ age‐specific annual deviation of established females (i.e. non‐recruits). Further details and parameter estimation for both vital rate functions are summarized in Appendix S2 (Supporting information). We assume that the mean survival of age *a* males is equal to that of females; that is, as males do not express the trait, their survival just depends on age. The density functions of males after one time step is then

(eqn 8) $$n_{m}^{\left(t+1\right)}\left(g,a+1\right)=s_{m}^{\left(t\right)}\left(a\right)n_{m}^{\left(t\right)}\left(g,a\right),$$

where $s_{m}^{\left(t\right)}\left(a\right)\;=\;\int \int s_{f}^{\left(t\right)}\left(x,g,a\right)n_{f}^{\left(t\right)}\left(x,g,a\right)dxdg/\int \int n_{f}^{\left(t\right)}\left(x,g,a\right)dxdg$.

The number of successful recruits derived from age *a* females (i.e. those surviving their first winter to breed at age one) and the distribution of the female recruits’ labile component of laying date at first breeding are denoted by $b_{f}^{\left(t\right)}\left(x,g,a\right)$ and $C^{\left(t\right)}\left(x^{\prime }\right)$, respectively. The recruitment function has the same basic structure as the survival model, with time‐varying second‐degree polynomial terms for laying date, second‐degree polynomial age terms, and linear breeding pair density and secular trend terms. The resulting $b_{f}^{\left(t\right)}\left(x,g,a\right)$ is density‐dependent. The recruit kernel for the labile component of laying date synchrony is modelled as a normal density function with conditional mean given by $\mu_{C}\;=\;\gamma_{1}^{\left(t+1\right)}$, where $\gamma_{1}^{\left(t+1\right)}$ is the stochastic annual deviation of new recruits. Further details and parameter estimation for both vital rate functions are summarized in Appendix S2 (Supporting information).

To calculate the density functions of new recruits, we need to construct the joint probability density function of offspring $x^{\prime }$ and $g^{\prime }$ states in females and the probability density function of $g^{\prime }$ in males. To do this, we first calculate the joint probability density function of maternal genotype and female recruit *l*‐state as

(eqn 9) $$\begin{array}{cc} \chi_{f}^{\left(t\right)}\left(g_{f},x^{\prime }\right) & =\frac{C^{\left(t\right)}\left(x^{\prime }\right)}{\Delta^{\left(t\right)}}\sum_{a}\int b_{f}^{\left(t\right)}\left(x,g,a\right)n_{f}^{\left(t\right)}\left(x,g,a\right)\;dx, \end{array}$$

where $\Delta^{\left(t\right)}$ is the normalization term, that is $\Delta^{\left(t\right)}\;=\;\sum_{a}\int \int b_{f}^{\left(t\right)}\left(x,g,a\right)n_{f}^{\left(t\right)}\left(x,g,a\right)dxdg$. Note that we define a single recruitment function $b_{f}$ for the production of male and female recruits, effectively assuming a 1:1 sex ratio and equal probability of male and female recruitment. The pdf of maternal genotype conditional on the *l*‐state in female offspring is then given by $\Phi_{f}^{\left(t\right)}\left(g_{f}|x^{\prime }\right)\;=\;\chi_{f}^{\left(t\right)}\left(g_{f},x^{\prime }\right)/\chi_{f}^{\left(t\right)}\left(x^{\prime }\right)$. As the focal trait is only expressed in females, the probability density function of maternal genotype among male recruits *l*‐state is calculated directly as

(eqn 10) $$\begin{array}{cc} \Phi_{m}^{\left(t\right)}\left(g_{f}\right) & =\frac{1}{\Delta^{\left(t\right)}}\sum_{a}\int b_{f}^{\left(t\right)}\left(x,g,a\right)n_{f}^{\left(t\right)}\left(x,g,a\right)\;dx, \end{array}$$

As our model assumes that male reproductive success is independent of their state (i.e. their reproductive success is independent of age), the probability density function of paternal genotype among female and male recruits is equal to the normalized paternal genotype density function, given by

(eqn 11) $$\Phi_{f}^{\left(t\right)}\left(g_{m}\right)=\Phi_{m}^{\left(t\right)}\left(g_{m}\right)=\frac{1}{\Pi^{\left(t\right)}}\sum_{a}n_{m}^{\left(t\right)}\left(g,a\right).$$

where $\Pi_{m}^{\left(t\right)}$ is the normalization term, $\Pi^{\left(t\right)}\;=\;\sum_{a}\int n_{m}^{\left(t\right)}\left(g,a\right)dg$. Once the (conditional) $\Phi^{\left(t\right)}$ are determined, the joint probability density function of offspring $x^{\prime }$ and $g^{\prime }$ states in females, $\Psi_{f}^{\left(t+1\right)}\left(x^{\prime },g^{\prime }\right)$, and the probability density function of $g^{\prime }$ in males, $\Psi_{m}^{\left(t+1\right)}\left(g^{\prime }\right)$, are straightforward to compute using the calculations described in Appendix S1. Once they have been calculated, we rescale $\Psi_{f}^{\left(t+1\right)}\left(x^{\prime },g^{\prime }\right)$ and $\Psi_{m}^{\left(t+1\right)}\left(g^{\prime }\right)$ by the total number of recruits of each sex, $N_{f}^{\left(t+1\right)}\left(0\right)$ and $N_{m}^{\left(t+1\right)}\left(0\right)$, to calculate the final density function of female and male recruits. As we assume that the sex ratio is 1:1, and average female and male survival to age 1 are equal, so that $N_{f}^{\left(t+1\right)}\left(0\right)$ = $N_{m}^{\left(t+1\right)}\left(0\right)$, which are just the normalization constant, $\Delta^{\left(t\right)}$, calculated above.

To implement the model, the integrations were approximated by the midpoint rule, using 50 nodes for both the *x* and *g* states. The stochastic environment component of the model was generated using a resampling approach – at each iteration we sampled the set of year effects associated with a randomly chosen year (*iid* sampling), the density of breeding pairs calculated, and then, these were used to construct the model components for that step. Each simulation was initialized with 100 individuals, and first run for 100 years with parent and offspring genotype uncoupled (i.e. using a purely ’ecological’ model). The complete model was implemented in R (R Core Team 2014).

### Decomposing annual change

We now describe a simple elaboration of the age‐structured Price equation that is appropriate for two‐sex systems subject to maternal demographic control (as assumed in our case study). The latter assumption considerably simplifies the development of the decomposition as it ensures that contributions to the next generation from breeding females and males are independent. The resulting decomposition partitions the annual change in the mean value of a state variable into components due to variation in demographic structure and sex ratio, age‐specific selection via differences in survival and recruitment, phenotypic plasticity and growth, and differences between offspring and parental states. The principal advantage of the ASPE is that it focusses on short time steps. This is convenient for comparing components of phenotypic change in species with overlapping generations, because it evaluates change on a common scale and separates change due to purely demographic processes from that occurring via selection, inheritance, plasticity and growth.

We describe the partition with respect to a general univariate state variable, denoted *y*, which may correspond to either the ‘*l*‐state’, the ‘*q*‐state’ or a component of the ‘*q*‐state’ such as the breeding value. Following Coulson, Tuljapurkar & Childs (2010), selection differentials in the decomposition are expressed in terms of differences among means, rather than covariances between components of fitness and the focal trait distribution. Female and male terms are denoted by the subscripts *f* and *m*, but we also use the ∘ subscript to denote *m* or *f* generically, to reduce repetition. The mean state of females and males of age *a* in the base population are ${\overset{¯}{y}}_{f}\left(a,t\right)$ and ${\overset{¯}{y}}_{m}\left(a,t\right)$, respectively. The corresponding mean state of age *a* individuals following selection on survival and recruitment are ${\overset{¯}{y}}_{\circ }^{S}\left(a,t\right)$ and ${\overset{¯}{y}}_{\circ }^{R}\left(a,t\right)$, respectively. These expectations are calculated with respect to the appropriate weighted density functions. For example, if we are decomposing the labile component of the laying date synchrony model, the female recruitment term, ${\overset{¯}{x}}_{f}^{R}\left(a,t\right)$, is given by

(eqn 12) $${\overset{¯}{x}}_{f}^{R}\left(a,t\right)=\frac{\int \int xb_{f}^{\left(t\right)}\left(x,g,a\right)n_{f}^{\left(t\right)}\left(x,g,a\right)\;dx\;dg}{\int \int b_{f}^{\left(t\right)}\left(x,g,a\right)n_{f}^{\left(t\right)}\left(x,g,a\right)\;dx\;dg}.$$

The mean state of age *a* individuals after ontogenetic [’(G)rowth’] or plasticity‐induced change is ${\overset{¯}{y}}_{\circ }^{G}\left(a,t\right)$, and the mean state among recruits [‘(O)ffspring’] derived from reproducing individuals is ${\overset{¯}{y}}_{\circ }^{O}\left(a,t\right)$. For example, the ‘growth’ term associated with the labile component is given by

(eqn 13) $${\overset{¯}{x}}_{f}^{G}\left(a,t\right)=\frac{\int \int \int x^{\prime }G_{f}^{\left(t\right)}\left(x^{\prime }|x,a\right)s_{f}^{\left(t\right)}\left(x,g,a\right)n_{f}^{\left(t\right)}\left(x,g,a\right)\;dx{}^{\prime }\;dx\;dg}{\int \int s_{f}^{\left(t\right)}\left(x,g,a\right)n_{f}^{\left(t\right)}\left(x,g,a\right)\;dx\;dg}.$$

Five additional terms need to be defined to account for demographic processes. The first, $\overset{¯}{w}(t)$, is the population fitness, given by $N^{\left(t+1\right)}/N^{\left(t\right)}$. The second, $q_{f}\left(t\right)$ and $q_{m}\left(t\right)$, are the proportions of females and males in the population. The third, $c_{f}\left(a,t\right)$ and $c_{m}\left(a,t\right)$, are the proportion of age *a* individuals *within* each sex. The fourth, ${\overset{¯}{S}}_{f}\left(a,t\right)$ and ${\overset{¯}{S}}_{m}\left(a,t\right)$, are the mean age‐specific survival rates of females and males. The fifth, ${\overset{¯}{R}}_{f}\left(a,t\right)$ and ${\overset{¯}{R}}_{m}\left(a,t\right)$, are the mean age‐specific rates of recruitment from breeding females and males.

With these definitions in hand, the change in the overall mean, $\Delta \overset{¯}{y}(t)$, of the focal state variable can be written as

$$\begin{array}{cc} \Delta \overset{¯}{y}(t) & =\sum^{\omega }\frac{q_{f}\left(t\right)c_{f}\left(a,t\right){\overset{¯}{S}}_{f}\left(a,t\right)}{\overset{¯}{w}(t)}[{\overset{¯}{y}}_{f}^{S}\left(a,t\right)-{\overset{¯}{y}}_{f}\left(a,t\right)]\;\text{survival selection}\\ & +\sum^{\omega }\frac{q_{f}\left(t\right)c_{f}\left(a,t\right)\overset{¯}{S_{f}}\left(a,t\right)}{\overset{¯}{w}(t)}[{\overset{¯}{y}}_{f}^{G}\left(a,t\right)-{\overset{¯}{y}}_{f}^{S}\left(a,t\right)]\;\text{'growth'}\\ & +\sum^{\omega }\frac{q_{f}\left(t\right)c_{f}\left(a,t\right)\overset{¯}{R_{f}}\left(a,t\right)}{\overset{¯}{w}(t)}[{\overset{¯}{y}}_{f}^{R}\left(a,t\right)-{\overset{¯}{y}}_{f}\left(a,t\right)]\;\text{recruitment selection}\\ & +\sum^{\omega }\frac{q_{f}\left(t\right)c_{f}\left(a,t\right)\overset{¯}{R_{f}}\left(a,t\right)}{\overset{¯}{w}(t)}[{\overset{¯}{y}}_{f}^{O}\left(a,t\right)-{\overset{¯}{y}}_{f}^{R}\left(a,t\right)]\;\text{'inheritance'}\\ & +\sum^{\omega -1}\Delta [q_{f}\left(t\right)c_{f}\left(a,t\right)]{\overset{¯}{y}}_{f}\left(a,t\right)-q_{f}\left(t\right)c_{f}\left(\omega ,t\right){\overset{¯}{y}}_{f}\left(\omega ,t\right)\;\text{demography: survival}\\ & +\sum^{\omega }\frac{q_{f}\left(t\right)c_{f}\left(a,t\right)\overset{¯}{R_{f}}\left(a,t\right)}{\overset{¯}{w}(t)}{\overset{¯}{y}}_{f}\left(a,t\right)\;\;\text{demography: recruitment}\\ & +\;\text{Corresponding Male Terms.} \end{array}$$

Here, $\Delta [q_{\circ }\left(t\right)c_{\circ }\left(a,t\right)]\;=\;q_{\circ }\left(t\;+\;1\right)c_{\circ }\left(a,t\;+\;1\right)-q_{\circ }\left(t\right)c_{\circ }\left(a,t\right)$, and *ω* is the maximum age an individual of either sex can reach. For brevity, we have only shown the female terms of the decomposition, although the full decomposition includes an equivalent male term for each female term shown here.

The modified version of the ASPE is very similar to the original proposed by Coulson & Tuljapurkar (2008). The only difference is that here we rescale the different contributions according to the sex ratio. The first two terms show how surviving females from extant cohorts contribute to changes in the mean. The first term (‘survival’) is a survival selection differential; it shows difference in survival associated with the focal variable contribute to a shift of the mean. The second term (‘growth’) shows how phenotypic plasticity and/or ontogeny alters the mean. The next two terms show how the addition of new recruits to the population contributes to changes in the mean. The third term (‘recruitment’) is the recruitment selection differential; it shows how differences in recruitment associated with the focal variable shift the mean. We adopt the term ‘recruitment selection’ in place of ‘fertility selection’ (Coulson, Tuljapurkar & Childs 2010) to emphasize that this contribution is a consequence of both the fertility of parents and the viability of offspring. Note that ’recruitment selection’ acts on the phenotype of parents. The fourth term (‘inheritance’) describes the mean difference between offspring and parental states. We retain the label ‘inheritance’ for consistency with previous work, but it is important to realize that this term will absorb changes due to parental effects and phenotypic plasticity in offspring. In all four cases, the components inside square brackets are the mean change associated with a given age class and the components outside the square brackets weight these potential contributions to the overall change by the appropriate demographic weights – the latter depend on age‐specific mean survival or recruitment and the age/sex composition of the population. The final two terms describe how purely demographic processes – differences in survival and recruitment that are not linked to the focal state – alter the mean, that is those changes caused by shifts in sex‐specific age structure under constant age‐dependent mean phenotypes, including the contributions from obligate death in the final age class and recruits in the first age class. The fifth term gives the contribution from differences in mean age‐specific survival rates, while the last term describes how differences in reproductive rates between age classes contribute to change.

## Results

**Figure 1:**
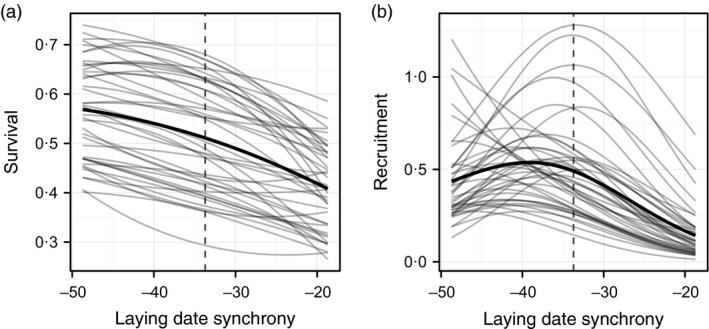
(a) Survival and (b) recruitment of females as a function of laying date synchrony in the Wytham great tit population, which is defined as the difference between mean first egg‐laying date and caterpillar half‐fall date. The thin grey lines show the annual fitness components and the thick black line shows the mean function. Vertical dashed line is the population mean value of laying date synchrony.

The modelled time‐varying associations between laying date synchrony and (a) survival and (b) recruitment are shown in Fig. 1. The survival associations are largely linear, and although mean survival varies among years, there is consistent directional selection for early breeding via this component of fitness. The recruitment associations are generally nonlinear and hump shaped, with substantial variation in mean recruitment evident, and the strength and direction of selection via this component of fitness vary from weakly positive in a few years to negative in others. Taken together, the survival and recruitment functions indicate that the population should evolve towards an earlier laying date, with an optimum value of the interval laying date and half‐fall date that is at least approximately 7–10 days larger than the current value. Once this optimum is reached, it appears that (stochastic) stasis will be maintained by antagonistic selection on recruitment and survival, that is later laying will generally favour recruitment but earlier laying increases survival.

**Figure 2:**
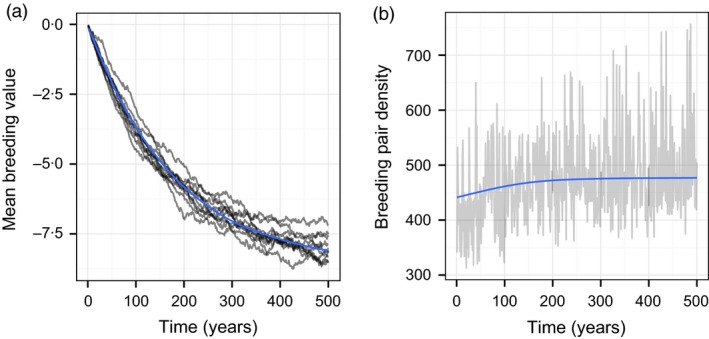
Time series showing predictions of (a) the mean breeding value of laying date synchrony, and (b) the density of breeding pairs in the modelled population. Simulations were carried out for 500 years. Blue lines show the mean value in each year, estimated from 250 independent simulations. Grey lines show representative results from 10 simulations (breeding values) or a single simulation (breeding pair density).

We used the model to track the expected change in population density and laying date synchrony. The expected mean trajectory of laying date synchrony – given as the change in the mean breeding value ($\overset{¯}{g}$) – and the change in breeding pair density are shown in Fig. 2. As expected, the model predicts that the interval between half‐fall date and egg‐laying date will increase by just over one week (Fig. 2a), although the predicted rate of change is slow; on average, a change of approximately 1 day is expected in the first 20 years. Although environmental stochasticity introduces a degree of uncertainty into this prediction, projections from independent simulations are largely consistent. Earlier laying is predicted to increase the survival of established individuals and the recruitment of offspring, and concomitant with the change in laying date synchrony, the model predicts an increase from about 440 to 480 breeding pairs on average (Fig. 2b).

**Figure 3:**
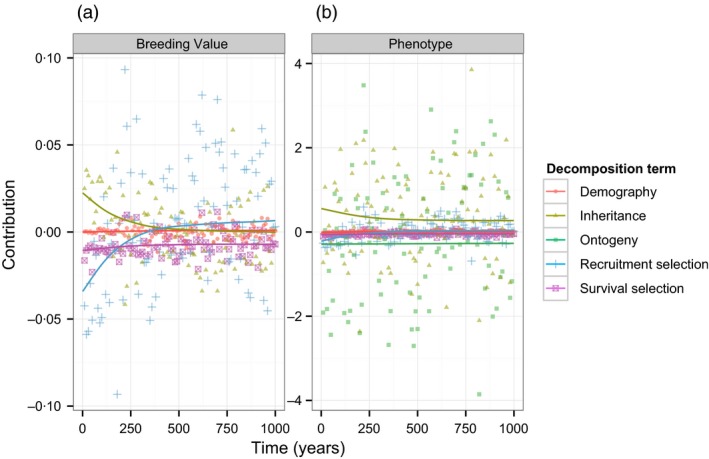
Predicted components of the age‐structured Price equation decomposition of the annual change in (a) the mean breeding value of laying date synchrony, and (b) the laying date synchrony phenotype, of female great tits in Wytham. Figures show these contributions change over 1000 years as the population evolves towards stasis. Lines show the mean value of each component of the decomposition taken with respect to 250 independent simulations. Points show the value of each component from a single representative simulation, in every 10th year.

Next, we applied the modified ASPE to the model to better understand the component drivers of change at both the phenotypic and genotypic levels. Figure 3 shows how the expected female contributions to annual change from each term change over time for (a) the mean breeding value, $\Delta \overset{¯}{g}(t)$, and (b) the mean phenotype, Δ[*x*(*t*)+*g*(*t*)], summed over age classes. The lines show the mean contribution calculated from 250 simulations, and the points show a subsample of annual contributions from a single representative simulation. Rather than separating the two demographic process terms, we chose to summarize their combined effect in Fig. 3.

In general, the different contributions to the change in the mean breeding value are very small (Fig. 3a), reflecting weak selection on the laying date synchrony near its optimum value and its low heritability in the model ($h^{2}\approx 0\cdot 16$). The survival selection component (purple line, squares/crosses) is consistently negative and exhibits relatively little variation among years. In contrast, the recruitment selection component (blue line, ‘+’ symbols) tends to be negative in the early phase of evolution and then positive, with much larger fluctuations overall. Selection acts antagonistically on recruitment and survival once stochastic, evolutionary stasis is reached. The mean inheritance contributions (yellow line, filled triangles) are initially positive – recruits have more positive breeding values than their mothers – and then decay to zero as stasis is reached. There is no ‘growth’ contribution associated with the mean breeding value, as this is invariant over an individual's lifetime. The aggregate demographic effect (red line, circles) is negligible, reflecting the fact that the mean trait value does not change much with age. The male inheritance term (not shown) is the only nonzero contribution to changes in the mean breeding value from males. This term is always exactly equal in magnitude, but opposite in sign, to that of females.

The annual contributions of different processes to changes in the mean phenotype (Fig. 3b) are larger than their breeding value counterparts, because they include the shared, stochastic component of annual variation. However, although they exhibit larger fluctuations, the temporal change in the recruitment and survival contributions is identical to those associated with the mean breeding value; the realized laying date synchrony is an additive function of breeding value and so any change in the latter is reflected in the phenotype. The aggregate demographic contributions to phenotypic changes are also very similar to the breeding value complements; these are very small, again reflecting the limited age structuring of vital rate and mean trait differences. The two largest terms are those due to inheritance and plasticity, both of which exhibit relatively large annual fluctuations. These terms are consistently nonzero (on average) even after evolutionary stasis is reached. The inheritance term is generally positive, such that new recruits tend to have later laying dates than established females, while the plasticity term is generally negative, indicating that individuals start egg‐laying earlier as they grow older. However, the magnitude of these average effects is modest relative to the scale of their annual fluctuations.

**Figure 4:**
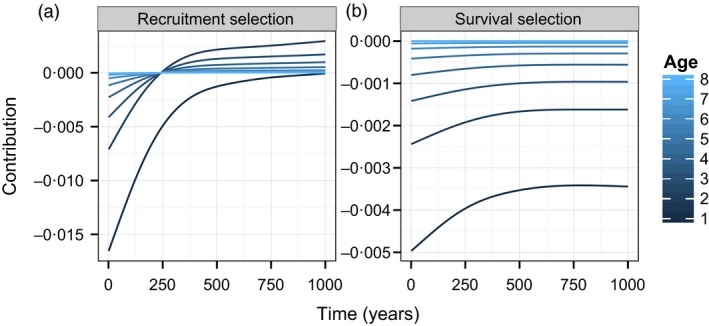
Age‐specific contributions from (a) recruitment selection and (b) survival selection to the age‐structured Price equation decomposition of the annual change in the mean breeding value of laying date synchrony. Lines show the mean value of each component of the decomposition taken with respect to 250 independent simulations, each run for 1000 years.

Finally, we examined the age‐specific component of the two selection terms associated with the mean breeding value (Fig. 4, lines show the mean contribution calculated from 250 simulations). Initially, there is selection for early laying date synchrony via recruitment differences (left panel). The largest negative contributions were from new recruits (age = 1), tending towards zero in older age classes. As stasis is reached, this pattern becomes hump shaped, such that these terms are near zero in new recruits and older individuals, and positive in intermediate age classes. This pattern reflects the opposing effects of an age‐dependent shift in mean and the decreasing demographic weights attached to older individuals. The age‐specific contributions due to survival selection are always negative. The age‐pattern is monotonic, such that the largest negative contributions are always from new recruits, reflecting the decreasing demographic weights attached to individuals as they age.

## Discussion

Long‐term, individual‐based studies of birds and mammals are now commonly used to explore the consequences of variation in life‐history, morphometric, behavioural and social traits [reviewed in Clutton‐Brock & Sheldon (2010)]. The framework described here has the potential to garner greater insight into the nature of selection and its consequences in such systems when focal traits vary over an individual's lifetime through ontogeny or phenotypic plasticity. We have shown how the micro‐evolutionary dynamics of labile traits and concomitant ecological change can be predicted by integrating quantitative trait information into data‐driven structured models. The resulting models are similar to standard IPMs, with many of the advantages this entails (Rees, Childs & Ellner 2014): (i) given adequate knowledge of the life cycle and careful demographic accounting, it is relatively straightforward to specify a data‐driven model for organisms with complex life histories; (ii) with sufficient longitudinal data describing individual performance and trait dynamics, each component function of the resulting model can be efficiently parameterized using common regression tools; (iii) the resulting models take a dynamic view of labile traits, accommodating patterns of expression that depend on abiotic and/or biotic components of the current and past environments; and (iv) as the response to selection is an emergent property, a model‐based analysis accounts for aspects of the biology that can be difficult to accommodate using standard selection analyses, such as sexual dimorphism in the life history or the expression of focal trait(s).

Integral projection models are increasingly being used to study contemporary evolutionary dynamics. Many such studies have focussed on putative eco‐evolutionary processes, where the dynamics of ecological and phenotypic change are thought to be mutually dependent and operate over similar time‐scales (Fussmann, Loreau & Abrams 2007; Pelletier, Garant & Hendry 2009; Ellner 2013). While there remains general agreement that IPMs are effective at capturing variation due to ontogeny and phenotypic plasticity, there is an emerging debate about the ability of such models to describe microevolutionary change (Hedrick *et al*. 2014; Traill, Schindler & Coulson 2014; Chevin 2015). In essence, these concerns derive from disagreement over the capacity of the ‘inheritance function’ ($C^{\left(t\right)}$, using our notation) to faithfully describe genetic transmission in an age‐structured population. Two related concerns are evident: (i) $C^{\left(t\right)}$ may confound the impact of genetic and parental effects on offspring phenotype when it is parameterized using parent–offspring regressions; and (ii) phenotypic associations between offspring and parents of different ages are a consequence of accumulated differences in the latter owing to individual variation in growth trajectories (Chevin 2015). Incorporating individual heterogeneity into IPMs addresses the latter criticism (Rees *et al*. 2000; Ellner & Rees 2006, 2007; Rees & Ellner 2016; Vindenes & Langangen 2015). Our framework takes this idea further by separating the focal traits/states into labile and permanent genetic/environmental components, which deals with the former concern.

In our case study, we used a form of retrospective analysis – the age‐structured Price equation – to partition sources of genetic and phenotypic change predicted by the model. We found that at equilibrium genetic stasis is maintained by a balance between antagonistic selection between the recruitment and survival components of fitness. This is not particularly surprising, given the form of the two fitness functions summarized in Fig. 1. However, the purely phenotypic analysis suggests that stasis additionally involves a balance between the ‘ontogeny’ and ‘inheritance’ contributions. This has nothing to do with the transmission of genes or trade‐offs among growth and reproduction. Instead, it reflects two processes. First, successive bouts of survival selection shift the mean towards earlier laying dates as a cohort ages – this shift is inflated by the autocorrelation in the labile component. Secondly, the model includes a fixed difference between laying date synchrony of new recruits and established females (supported by the data), whereby established females tend to lay earlier than recruits. This difference might be a result of experienced females outperforming inexperienced recruits, or alternatively, it may reflect our failure during the model parameterization step to account for selection removing ‘low‐quality’ individuals (Bouwhuis *et al*. 2009).

As our goal here was to illustrate the methods and their uses, we adopted a very simple model of laying date synchrony. A more mechanistic approach would be to model the true lay date and half‐fall date as functions of spring temperature, although this would need to include a submodel to project future temperature changes. Moreover, although we have explored the dynamics of the trait with reference to population‐scale variation, in the present case the synchrony phenotype may show important scale‐dependent effects. As the effective foraging range of parental birds is quite limited, spatial variation in the phenology of caterpillar timing, which may be driven in turn by spatial, or individual‐level, variability in tree phenology may select for different optima for different parts of the population. Indeed, there is evidence to suggest that breeding phenology of great tits is partially predicted by very small‐scale synchrony with their local environment (Cole *et al*. 2015; Hinks *et al*. 2015). In principle, these kinds of mechanisms could be explored using a spatial extension of our model.

Theoretical studies are beginning to reveal the general conditions that favour the evolution of quantitative, labile trait variation (Lande 2014). Evaluating the adaptive significance of labile traits in animal populations is complicated by factors such as sexual dimorphism in life histories, state‐dependent vital rate variation, and density dependence. The benefit of constructing a demographic projection model is that it can be used as a tool to not only project, but also to understand the processes that alter trait distributions, population structure and density. Many tools devised to analyse matrix and integral projection models can be applied to our framework. For example, summary statistics of demography such as cohort‐specific generation time, lifespan or age at first reproduction can be calculated, and perturbation analyses can then be used to understand how these summary statistics respond to changes in the underlying vital rates (Coulson 2012; Smallegange & Coulson 2013). Nonetheless, although it can accommodate many of the complexities associated with ’realistic’ life histories, our model‐based framework is still subject to some of the same limitations that afflict phenotypic selection analysis of natural populations. Crucially, it assumes that estimated fate–phenotype relationships are causal (Morrissey, Kruuk & Wilson 2010). For example, our case study assumes that differences in survival and recruitment are a direct consequence of their modelled associations with laying date synchrony (summarized in Fig. 1), yet these may in reality be driven by unmeasured factors such as differences in body condition, local density or nest site quality (Wilkin *et al*. 2006; Browne *et al*. 2007). A second practical limitation of the framework is that it can be computationally expensive to implement, requiring multidimensional integrals to be numerically evaluated at each iteration. In practice, this effectively restricts its application to situations where a small number of traits need to be modelled.

Quantitative genetic analyses of selection on ontogenetic trajectories typically proceed by treating the focal trait and associated breeding values as a multivariate, age‐dependent quantity (Kirkpatrick, Lofsvold & Bulmer 1990; Kirkpatrick & Lofsvold 1992), or by adopting a random regression description of the age–trait relationship (Wilson, Kruuk & Coltman 2005). These descriptions can be accommodated by our model, although they may not be optimal in a fluctuating environment. A key component of every IPM is the ’growth’ kernel, $G^{\left(t\right)}\left(x^{\prime }|\;x,\ldots \right)$, which, for ontogenetic traits, is derived from a first‐order antedependence model fitted by regressing the successive states against one‐another (as discussed in the Parameterization section). Under this model, the *l*‐state variable corresponds directly to the trait of interest and the *q*‐state captures heritable differences in the rate of state change, conditional on current state. Similar models have been previously been applied to the genetic analysis of cumulative traits such as body size (Jaffrézic *et al*. 2004). The advantage of such models is that they propagate the cumulative effect of past environments; ‘growth’ at each transition is conditioned on the current state, which is itself a consequence of past environments. The framework described here offers a way to integrate such analyses into demographic models to predict the microevolutionary consequences of ontogenetic trait variation.

The general model we have outlined here provides a useful framework for predicting microevolutionary dynamics of labile traits in natural populations, where longitudinal data describing trait, life history and vital rate variation have been collected. A key advantage of this approach over standard selection analyses is that it can be used to project evolutionary change using all the vital rates and genetic variance, which is otherwise difficult to do in structured populations subject to environmental stochasticity and density dependence (Brommer *et al*. 2004; Childs *et al*. 2011). When coupled with well‐developed tools from evolutionary demography, this will facilitate greater insight into processes that govern ecological and evolutionary change. However, in its most general form, this model is probably too complex for analytical results to be derived, although it may be possible to derive important results for special cases. For example, by assuming that vital rates are invariant with respect to time and sex, Barfield, Holt & Gomulkiewicz (2011) were able to show that Lande's theorem applies to discrete stage‐classified populations. Similar derivations may be possible for labile trait‐structured populations subject to these same constraints. Such efforts are clearly needed, as they will foster stronger links between theoretical and empirical studies of the adaptive significance of trait variation.

## Data accessibility

Data are available from the Dryad Digital Repository: http://dx.doi.org/10.5061/dryad.np084 (Childs et al. 2016).

## Supporting information


**Appendix S1.** Additional calculations and cases.
**Appendix S2.** Case study.
