# Supplementary material for: The evolution of labile traits in sex‐ and age‐structured populations
Source: J Anim Ecol. 2016 Feb 22;85(2):329–42. doi: 10.1111/1365-2656.12483 (PMC4768649; doi:10.1111/1365-2656.12483)
Supplement: Supplementary file 1 — Appendix S1. Additional calculations and cases. Appendix S2. Case study. [file JANE-85-329-s001.pdf]

Supporting Information:

The Evolution of Labile Traits in Sex- and Age-Structured Populations

Dylan Z. Childs, Ben Sheldon and Mark Rees

A Additional Calculations and Cases

A.1 Computing the joint offspring l- and q-state pdf

In this section we show how to calculate the l- and q-state probability density function of female and male recruits, given the joint pdf of their own l-state and their parents' genotype, denoted $\chi_f^{(t)}(\mathbf{g}_f, \mathbf{g}_m, \mathbf{x}')$ and $\chi_m^{(t)}(\mathbf{g}_f, \mathbf{g}_m, \mathbf{x}')$. We begin by calculating the joint pdf of maternal and paternal genotype among recruits, conditional on recruit l-states. These are simply

$$\Phi_f^{(t)}(\mathbf{g}_f, \mathbf{g}_m|\mathbf{x}') = \frac{\chi_f^{(t)}(\mathbf{g}_f, \mathbf{g}_m, \mathbf{x}')}{\chi_f^{(t)}(\mathbf{x}')} \tag{A.1}$$

$$\text{and} \quad \Phi_m^{(t)}(\mathbf{g}_f, \mathbf{g}_m|\mathbf{x}') = \frac{\chi_m^{(t)}(\mathbf{g}_f, \mathbf{g}_m, \mathbf{x}')}{\chi_m^{(t)}(\mathbf{x}')}, \tag{A.2}$$

where $\Phi_f^{(t)}(\mathbf{g}_f, \mathbf{g}_m, |\mathbf{x}')$ and $\Phi_m^{(t)}(\mathbf{g}_f, \mathbf{g}_m, |\mathbf{x}')$ are the required conditional pdfs in female and male recruits, respectively. The terms $\chi_f(\mathbf{x}')$ and $\chi_m(\mathbf{x}')$ are the corresponding marginal l-state pdfs. That is, $\chi_f(\mathbf{x}') = \iint \chi_f^{(t)}(\mathbf{g}_f, \mathbf{g}_m, \mathbf{x}')\, d\mathbf{g}_f\, d\mathbf{g}_m$ and $\chi_m(\mathbf{x}') = \iint \chi_m^{(t)}(\mathbf{g}_f, \mathbf{g}_m, \mathbf{x}')\, d\mathbf{g}_f\, d\mathbf{g}_m$. With these distributions in hand we can then calculate the conditional pdf associated with the midparent values ($\hat{\mathbf{g}}$) in female and male recruits, denoted $\Gamma_f^{(t)}(\hat{\mathbf{g}}|\mathbf{x}')$ and $\Gamma_m^{(t)}(\hat{\mathbf{g}}|\mathbf{x}')$, respectively. These are given by

$$\begin{aligned} \Gamma_f^{(t)}(\hat{\mathbf{g}}|\mathbf{x}') &= \int \Phi_f^{(t)}\left(\mathbf{g}, 2\hat{\mathbf{g}} - \mathbf{g} \;\middle|\; \mathbf{x}'\right) d\mathbf{g} \\ \text{and} \quad \Gamma_m^{(t)}(\hat{\mathbf{g}}|\mathbf{x}') &= \int \Phi_m^{(t)}\left(\mathbf{g}, 2\hat{\mathbf{g}} - \mathbf{g} \;\middle|\; \mathbf{x}'\right) d\mathbf{g}\,. \end{aligned} \tag{A.3}$$

If parental genotypes do not covary among recruits, then equations A.3 reduce to a pair of convolutions (see next section). The conditional pdf of genotypes among female and male recruits, $\Omega_f^{(t)}(\mathbf{g}'|\mathbf{x}')$ and $\Omega_m^{(t)}(\mathbf{g}'|\mathbf{x}')$, are found by convolution with a (multivariate) normal pdf describing the segregation variance. That is

$$\begin{aligned} \Omega_f^{(t)}(\mathbf{g}'|\mathbf{x}') &= \int f_A\left(\mathbf{g}' - \hat{\mathbf{g}}\right) \Gamma_f^{(t)}(\hat{\mathbf{g}}|\mathbf{x}')\, \mathrm{d}\hat{\mathbf{g}} \\ \text{and} \quad \Omega_m^{(t)}(\mathbf{g}'|\mathbf{x}') &= \int f_A\left(\mathbf{g}' - \hat{\mathbf{g}}\right) \Gamma_m^{(t)}(\hat{\mathbf{g}}|\mathbf{x}')\, \mathrm{d}\hat{\mathbf{g}}, \end{aligned} \tag{A.4}$$

where f_A is the pdf of a multivariate normal distribution with mean $\mathbf{0}$ and (co)variance $\mathbf{V}_A/2$. Finally, assuming environmental deviations are multivariate normal and independent of offspring state, the joint l- and q-state pdfs in female and male recruits, $\Psi_f^{(t+1)}(\mathbf{x}', \mathbf{z}', \mathbf{g}')$ and $\Psi_m^{(t+1)}(\mathbf{x}', \mathbf{z}', \mathbf{g}')$, are given by

$$\begin{aligned} \Psi_f^{(t+1)}(\mathbf{x}', \mathbf{z}', \mathbf{g}') &= f_E(\mathbf{z}' - \mathbf{g}')\Omega_f^{(t)}(\mathbf{g}'|\mathbf{x}')\chi_f^{(t)}(\mathbf{x}') \\ \text{and} \quad \Psi_m^{(t+1)}(\mathbf{x}', \mathbf{z}', \mathbf{g}') &= f_E(\mathbf{z}' - \mathbf{g}')\Omega_m^{(t)}(\mathbf{g}'|\mathbf{x}')\chi_m^{(t)}(\mathbf{x}'), \end{aligned} \tag{A.5}$$

where f_E is the pdf of a multivariate normal distribution with mean $\mathbf{0}$ and (co)variance $\mathbf{V}_E$.

A.2 Maternal demographic control

In this section we consider a situation we refer to as “maternal demographic control”. By this we mean that both the number of recruits produced and the offspring sex ratio are strictly determined by breeding females. This means, for example, that female reproduction is never limited by the availability of males. We also assume females may mate with more than one male, but that they do not have control over which males they mate with. Mate choice is therefore random with respect to female and male l- and q-states, although the relative reproductive success of individuals of each sex may be influenced by their state. The model is therefore appropriate for situations where reproduction occurs via a lottery. Two examples of situations where such a model might be appropriate are: 1) aquatic organisms employing open substrate spawning and 2) reproduction during the rut in certain ungulates.

The number of female and male recruits produced by a female are described by fertility functions $b_{f\to f}^{(t)}(\mathbf{x}, \mathbf{z}, a)$ and $b_{f\to m}^{(t)}(\mathbf{x}, \mathbf{z}, a)$, respectively. The relative reproductive success of a

male is determined the fertility function $b_m^{(t)}(\mathbf{x}, \mathbf{z}, a)$. Note that if male reproductive success is modelled as a state-dependent (i.e. weighted) lottery, then $b_m^{(t)}(\mathbf{x}, \mathbf{z}, a)$ will be a function of $n_m^{(t)}(\mathbf{x}, \mathbf{z}, \mathbf{g}, a)$. The joint probability density function of maternal genotype, paternal genotype and female offspring l-state in female and male offspring, $\chi_f^{(t)}(\mathbf{g}_f, \mathbf{g}_m, \mathbf{x}')$ and $\chi_m^{(t)}(\mathbf{g}_f, \mathbf{g}_m, \mathbf{x}')$, are then given by

$$
\begin{aligned}
\chi_f^{(t)}(\mathbf{g}_f, \mathbf{g}_m, \mathbf{x}') = \frac{1}{\Delta_f^{(t)}} \sum_{a_f, a_m} \iint & b_{f\to f}^{(t)}(\mathbf{x}, \mathbf{z}, a) b_m^{(t)}(\mathbf{x}, \mathbf{z}, a) \\
& \times C_{\to f}^{(t)}(\mathbf{x}' | \langle \mathbf{x}, \mathbf{z}, a\rangle_f, \langle \mathbf{x}, \mathbf{z}, a\rangle_m) \\
& \times n_f^{(t)}(\mathbf{x}, \mathbf{z}, \mathbf{g}, a) n_m^{(t)}(\mathbf{x}, \mathbf{z}, \mathbf{g}, a)\, \mathrm{d}\mathbf{x}\, \mathrm{d}\mathbf{z} \\
\text{and} \quad \chi_m^{(t)}(\mathbf{g}_f, \mathbf{g}_m, \mathbf{x}') = \frac{1}{\Delta_m^{(t)}} \sum_{a_f, a_m} \iint & b_{f\to m}^{(t)}(\mathbf{x}, \mathbf{z}, a) b_m^{(t)}(\mathbf{x}, \mathbf{z}, a) \\
& \times C_{\to m}^{(t)}(\mathbf{x}' | \langle \mathbf{x}, \mathbf{z}, a\rangle_f, \langle \mathbf{x}, \mathbf{z}, a\rangle_m) \\
& \times n_f^{(t)}(\mathbf{x}, \mathbf{z}, \mathbf{g}, a) n_m^{(t)}(\mathbf{x}, \mathbf{z}, \mathbf{g}, a)\, \mathrm{d}\mathbf{x}\, \mathrm{d}\mathbf{z}\,.
\end{aligned} \tag{A.6}
$$

Again, $\Delta_f^{(t)}$ and $\Delta_m^{(t)}$ are the normalisation terms corresponding to the integrands in equations A.6. A useful consequence of assuming random mating and female demographic control is that parental genotypes do not covary among offspring. Therefore, we can work with the conditional probability density functions of each parental sex separately to determine the distribution of midparent values. For each offspring sex, we write the pdf of each parental genotype, conditional on the offspring l-state. These are

$$
\begin{aligned}
\Phi_f^{(t)}(\mathbf{g}_f | \mathbf{x}') = \frac{\int \chi_f^{(t)}(\mathbf{g}_f, \mathbf{g}_m, \mathbf{x}') \mathrm{d}\mathbf{g}_m}{\chi_f^{(t)}(\mathbf{x}')}, \ \Phi_f^{(t)}(\mathbf{g}_m | \mathbf{x}') = \frac{\int \chi_f^{(t)}(\mathbf{g}_f, \mathbf{g}_m, \mathbf{x}') \mathrm{d}\mathbf{g}_f}{\chi_f^{(t)}(\mathbf{x}')}, \\
\Phi_m^{(t)}(\mathbf{g}_f | \mathbf{x}') = \frac{\int \chi_m^{(t)}(\mathbf{g}_f, \mathbf{g}_m, \mathbf{x}') \mathrm{d}\mathbf{g}_m}{\chi_m^{(t)}(\mathbf{x}')}, \ \Phi_m^{(t)}(\mathbf{g}_m | \mathbf{x}') = \frac{\int \chi_m^{(t)}(\mathbf{g}_f, \mathbf{g}_m, \mathbf{x}') \mathrm{d}\mathbf{g}_f}{\chi_m^{(t)}(\mathbf{x}')},
\end{aligned} \tag{A.7}
$$

where, for example, $\Phi_f^{(t)}(\mathbf{g}_m, | \mathbf{x}')$ is the conditional pdf of male parent genotypes among female offspring. The terms $\chi_f^{(t)}(\mathbf{x}')$ and $\chi_m^{(t)}(\mathbf{x}')$ are are the corresponding marginal l-state pdfs. The conditional offspring genotype distribution can then found by convolution, such that

$$
\begin{aligned}
\Omega_f^{(t)}(\mathbf{g}' | \mathbf{x}') = \Phi_f^{(t)}\left(\frac{\mathbf{g}_f}{2} | \mathbf{x}'\right) * \Phi_f^{(t)}\left(\frac{\mathbf{g}_m}{2} | \mathbf{x}'\right) * f_A(\mathbf{g}) \\
\text{and} \quad \Omega_m^{(t)}(\mathbf{g}' | \mathbf{x}') = \Phi_m^{(t)}\left(\frac{\mathbf{g}_f}{2} | \mathbf{x}'\right) * \Phi_m^{(t)}\left(\frac{\mathbf{g}_m}{2} | \mathbf{x}'\right) * f_A(\mathbf{g})\,,
\end{aligned} \tag{A.8}
$$

where f_A is the pdf of a multivariate normal defined above. Exactly as before, the joint l- and q-state probability density functions in new recruits of each sex are given by

$$\begin{aligned} &\Psi_f^{(t)}(\mathbf{x}', \mathbf{z}', \mathbf{g}') = f_E(\mathbf{z}' - \mathbf{g}')\Omega_f^{(t)}(\mathbf{g}'|\mathbf{x}')\Gamma_f^{(t)}(\mathbf{x}') \\ \text{and} \quad &\Psi_m^{(t)}(\mathbf{x}', \mathbf{z}', \mathbf{g}') = f_E(\mathbf{z}' - \mathbf{g}')\Omega_m^{(t)}(\mathbf{g}'|\mathbf{x}')\Gamma_m^{(t)}(\mathbf{x}'), \end{aligned} \tag{A.9}$$

where f_E is the pdf of a multivariate normal defined above. In order to compute the density function of new recruits we need to rescale $\Psi_f^{(t)}(\mathbf{x}', \mathbf{z}', \mathbf{g}')$ and $\Psi_m^{(t)}(\mathbf{x}', \mathbf{z}', \mathbf{g}')$ by the total abundance of female and male recruits, respectively. Recruitment is controlled by females. Therefore, these abundances depend only on $b_{f\to f}^{(t)}(\ldots)$ and $b_{f\to m}^{(t)}(\ldots)$, such that

$$\begin{aligned} n_f^{(t+1)}(\mathbf{x}', \mathbf{z}', \mathbf{g}', 0) &= \Psi_f^{(t)}(\mathbf{x}', \mathbf{z}', \mathbf{g}') \sum_{a_f} \iiint b_{f\to f}^{(t)}(\mathbf{x}, \mathbf{z}, a) n_f^{(t)}(\mathbf{x}, \mathbf{z}, \mathbf{g}, a)\, \mathrm{d}\mathbf{x}\mathrm{d}\mathbf{z}\mathrm{d}\mathbf{g} \\ n_m^{(t+1)}(\mathbf{x}', \mathbf{z}', \mathbf{g}', 0) &= \Psi_m^{(t)}(\mathbf{x}', \mathbf{z}', \mathbf{g}') \sum_{a_f} \iiint b_{f\to m}^{(t)}(\mathbf{x}, \mathbf{z}, a) n_f^{(t)}(\mathbf{x}, \mathbf{z}, \mathbf{g}, a)\, \mathrm{d}\mathbf{x}\mathrm{d}\mathbf{z}\mathrm{d}\mathbf{g} \end{aligned} . \tag{A.10}$$

A.3 Maternal phenotypic (and demographic) control

The previous model assumed that recruit production and offspring sex ratio are strictly determined by breeding females, but that the offspring l-state is jointly determined by the state of both parents. That is, both maternal and paternal effects are important. In many situations it may be more natural to assume that in the presence of maternal demographic control only maternal effects are operating. We refer to the latter situation as "maternal phenotypic control". In this section we simplify the previous model to consider the case where both types of maternal control operate. To do this, we redefine the recruit l-state kernels to be functions of only maternal state, such that the female and male offspring l-state kernels are now denoted $C_{\to f}^{(t)}(\mathbf{x}'|\langle\mathbf{x}, \mathbf{z}, a\rangle_f)$ and $C_{\to m}^{(t)}(\mathbf{x}'|\langle\mathbf{x}, \mathbf{z}, a\rangle_f)$, respectively. We retain the $\langle\ldots\rangle$ notation for consistency even though it is now redundant.

The assumption of maternal phenotypic and demographic control means that: 1) parental genotypes do not covary among offspring (maternal demographic control) and 2) paternal genotype and offspring l-state do not covary (maternal phenotypic control). Therefore, we can consider male and female contributions to the next generation separately. To begin, we calculate the joint probability density function of maternal genotype and recruit l-state in female and male offspring,

$\chi_f^{(t)}(\mathbf{g}_f, \mathbf{x}')$ and $\chi_m^{(t)}(\mathbf{g}_f, \mathbf{x}')$. These are then given by

$$
\begin{aligned}
\chi_f^{(t)}(\mathbf{g}_f, \mathbf{x}') &= \frac{1}{\Delta_f^{(t)}} \sum_{a_f} \iint b_{f\to f}^{(t)}(\mathbf{x}, \mathbf{z}, a) C_{\to f}^{(t)}(\mathbf{x}' | \langle \mathbf{x}, \mathbf{z}, a \rangle_f) \\
&\qquad \times n_f^{(t)}(\mathbf{x}, \mathbf{z}, \mathbf{g}, a)\, \mathrm{d}\mathbf{x}\, \mathrm{d}\mathbf{z} \\
\text{and} \quad \chi_m^{(t)}(\mathbf{g}_f, \mathbf{x}') &= \frac{1}{\Delta_m^{(t)}} \sum_{a_f} \iint b_{f\to m}^{(t)}(\mathbf{x}, \mathbf{z}, a) C_{\to m}^{(t)}(\mathbf{x}' | \langle \mathbf{x}, \mathbf{z}, a \rangle_f) \\
&\qquad \times n_f^{(t)}(\mathbf{x}, \mathbf{z}, \mathbf{g}, a)\, \mathrm{d}\mathbf{x}\, \mathrm{d}\mathbf{z},
\end{aligned}
\tag{A.11}
$$

where $\Delta_f^{(t)}$ and $\Delta_m^{(t)}$ are the normalisation terms corresponding to the integrands in equations A.11. The pdf of maternal genotype, conditional on the female and male recruit l-state are now

$$
\Phi_f^{(t)}(\mathbf{g}_f | \mathbf{x}') = \frac{\chi_f^{(t)}(\mathbf{g}_f, \mathbf{x}')}{\chi_f^{(t)}(\mathbf{x}')} \quad \text{and} \quad \Phi_m^{(t)}(\mathbf{g}_f | \mathbf{x}') = \frac{\chi_m^{(t)}(\mathbf{g}_f, \mathbf{x}')}{\chi_m^{(t)}(\mathbf{x}')}, \tag{A.12}
$$

where $\chi_f^{(t)}(\mathbf{x}')$ and $\chi_m^{(t)}(\mathbf{x}')$ are the corresponding marginal l-state pdfs. The paternal genotype pdf among female and male recruits are identical, and because paternal genotype and offspring l-state do not covary these can be calculated directly from the $b_m^{(t)}$ and $n_m^{(t)}$, such that

$$
\Phi_f^{(t)}(\mathbf{g}_m) = \Phi_m^{(t)}(\mathbf{g}_m) = \frac{1}{\Pi^{(t)}} \sum_a \iint b_m^{(t)}(\mathbf{x}, \mathbf{z}, a) n_m^{(t)}(\mathbf{x}, \mathbf{z}, \mathbf{g}, a)\, \mathrm{d}\mathbf{x}\, \mathrm{d}\mathbf{z} \tag{A.13}
$$

where $\Pi^{(t)}$ is the normalisation terms corresponding to the integrands in equation A.13. The conditional offspring genotype distribution can then be found by convolution, such that

$$
\begin{aligned}
\Omega_f^{(t)}(\mathbf{g}' | \mathbf{x}') &= \Phi_f^{(t)}\left(\frac{\mathbf{g}_f}{2} | \mathbf{x}'\right) * \Phi_f^{(t)}\left(\frac{\mathbf{g}_m}{2}\right) * f_A(\mathbf{g}) \\
\text{and} \quad \Omega_m^{(t)}(\mathbf{g}' | \mathbf{x}') &= \Phi_m^{(t)}\left(\frac{\mathbf{g}_f}{2} | \mathbf{x}'\right) * \Phi_m^{(t)}\left(\frac{\mathbf{g}_m}{2}\right) * f_A(\mathbf{g}),
\end{aligned}
\tag{A.14}
$$

where f_A is the pdf of a multivariate normal defined above. The remaining calculations need to derive the joint recruit l- and q-state distributions are given by equations A.9 and A.10.

B Case Study

B.1 Data analysis and parameter estimation

We analysed data from the period 1965-2007. The availability of breeding sites has been constant since 1964, and over this period every breeding attempt has been monitored from the date of first egg laying until all nestlings fledge. The size of most clutches are known, and at 15 days old, nestlings are marked with aluminium rings and parents are captured in order to determine their identity. The resulting dataset provides information about the relatedness, breeding phenology, and relative rates of recruitment success and adult survival. Rare second clutches and repeat clutches laid following the failure of the first clutch were excluded from the dataset. Demographic data from 3837 breeding attempts of females of known age were used to parameterise the model.

Four component functions are required to parameterise a minimal population model. The first two describe the survival of breeding females and recruitment of offspring into the breeding population. These were fitted as Binomial and Poisson mixed models with random year effects, respectively. Apparent survival (P_i) and recruitment (R_i) contributions from individual i in year t were modelled. Although it is known that capture rates are age-dependent (Bouwhuis et al., 2012), for simplicity, a breeding female was considered to have survived if it was observed breeding again in the following season, and the recruitment was determined from the number of ringed fledglings seen breeding at age one. The same model structure was used in both regressions. The fixed effects include a second degree polynomial terms for laying date synchrony (S_i) and age (A_i), along with linear density (N_t) and secular trend ('time', t) terms. Random effects for the intercept and slope terms associated with laying date synchrony were included to allow both the linear and non-linear components of selection to vary among years. The resulting model structure was:

$$g(E[X_{it}]) = \alpha_{\circ 0}^{(t)} + \alpha_{\circ 1}^{(t)} S_i + \alpha_{\circ 2}^{(t)} S_{it}^2 + \beta_{\circ 1} a_{it} + \beta_{\circ 2} a_{it}^2 + \delta_{\circ 1} N_t + \delta_{\circ 2} t, \tag{B.1}$$

where $\circ$ denotes either P (survival) or R (recruitment), $E[X_{it}]$ is the associated expectation, and g is the corresponding *logit* or *log* link function, respectively; $\alpha_0^{(t)}$ is the time-varying intercept; $\alpha_1^{(t)}$ and $\alpha_2^{(t)}$ are the time-varying polynomial coefficients with respect to synchrony; $\beta_1^{(t)}$ and $\beta_2^{(t)}$ are the fixed polynomial coefficients with respect to age; and $\delta_1^{(t)}$ and $\delta_2^{(t)}$ capture the fixed effects of density and time. All covariates were partially centred in both models, which were then fitted

using the `lme4` package (Bates et al., 2015) in R.

There was evidence for a significant linear effect of laying date ($\chi^2 = 9.90$, d.f. $= 1$, p < 0.01) on survival, though the quadratic effect ($\chi^2 = 0.31$, d.f. $= 1$, p $= 0.57$) was not significant. Nonetheless, we retained the fixed quadratic term because the model contained the corresponding random interaction with year. The quadratic effect of age on survival was significant ($\chi^2 = 4.09$, d.f. $= 1$, p < 0.05), and so we did not examine the linear effect. There was a significant secular trend in survival ($\chi^2 = 5.72$, d.f. $= 1$, p < 0.05), though the density term was marginal ($\chi^2 = 3.57$, d.f. $= 1$, p $= 0.06$). The quadratic effect ($\chi^2 = 15.1$, d.f. $= 1$, p < 0.001) of laying date on recruitment was significant, and so we did not examine the linear effect. Neither the quadratic effect ($\chi^2 = 0.05$, d.f. $= 1$, p $= 0.82$) or linear effect ($\chi^2 = 0.02$, d.f. $= 1$, p $= 0.90$) of age on recruitment were significant and so these were dropped from the model. There was a significant effect of density ($\chi^2 = 8.56$, d.f. $= 1$, p < 0.001) on recruitment, though the secular trend term was marginal ($\chi^2 = 3.58$, d.f. $= 1$, p $= 0.06$).

The mean values of the coefficients of the resulting survival model were: $\alpha_{P0} = 0.045$, $\alpha_{P1} = -0.022$, $\alpha_{P2} = -0.00041$, $\beta_{P1} = -0.024$, $\beta_{P2} = -0.047$, $\delta_{P1} = -0.0021$, and $\delta_{P2} = 0.029$. The mean values of the coefficients of the resulting recruitment model were: $\alpha_{R0} = 0.072$, $\alpha_{R1} = -0.044$, $\alpha_{R2} = -0.0035$, $\delta_{R1} = -0.0037$, and $\delta_{R2} = 0.025$. The intercept coefficients ($\alpha_{\circ 0}$) given here are with respect to the centred covariates model. The centring values used were: 0.34, with respect to S_i; 2.0, with respect to a_{it}; 240, with respect to N_t; and 2000, with respect to t. The year-specific values of the time-varying terms can be extracted from the R code and data that accompanies this manuscript.

The second two component functions of the population model describe the distribution of female laying date synchrony at recruitment, and the over subsequent ages. These are derived from a single animal model, fitted in two steps. We initially fitted a model that partitions the variance in synchrony attributable years, nest boxes, natal environment, permanent environment effects and additive genetic effects, assuming *iid* residuals. The estimated heritability of laying date synchrony derived from this model was 0.16. The only fixed effect included in the model was an variable indicating whether an observation was a new recruit. We then fitted a simpler model excluding every random effect other than the additive genetic variance term–which was fixed at the value estimated by the first model–but estimated the autocorrelation between residuals.

This approach estimates additive genetic variance as accurately as possible by conditioning on known sources of environmental variance, but captures the effect of the latter phenomenologically through a single term, the residual autocorrelation. The resulting model structure is:

$$S_{it} = \gamma_0^{(t)} + \gamma_1 A_{it} + g_i + x_{it}, \tag{B.2}$$

where $\gamma_0^{(t)}$ is the time-varying intercept, γ_1 is the coefficient for the age effect (new recruit or not), A_{it} is an indicator variable for whether or not $a_{it} > 1$, g_i is the breeding value, and x_{it} is the autocorrelated residual, with correlation coefficient ρ. The model was fitted in using ASREML. The mean values of the coefficients of the resulting model were: $\gamma_0 = 1.68$ and $\gamma_1 = -2.80$. The estimated standard deviation of the variance components associated with g_i and x_{it} were 2.13 and 4.88, respectively, and the magnitude of the autocorrelation (ρ) was 0.34. The year-specific values of the time-varying terms can be extracted from the R code and data that accompanies this manuscript.

B.2 Model calibration

The Wytham population is not closed. Consequently, our analysis of apparent survival and recruitment underestimates the true survival and recruitment rates. We calibrated the population model to account for this bias by adjusting the values of the survival and recruitment function intercepts to minimise a ‘loss function’, calculated as follows. For a given pair of intercept values, we simulated the population dynamics—decoupling parental and offspring breeding values so that the model was purely ‘ecological’—using a random set of 50 years followed by the observed sequence of year effects. We then calculated the mean square error of observed and predicted breeding pair densities over the observed year sequence. The survival and recruitment intercepts values that minimise this error were then adopted in our analysis of the Wytham population.
